# Neuromuscular development in the emerging scyphozoan model system, *Cassiopea xamachana*: implications for the evolution of cnidarian nervous systems

**DOI:** 10.3389/fnins.2023.1324980

**Published:** 2024-01-11

**Authors:** Klara Amplatz, Elisabeth Zieger, Daniel Abed-Navandi, Anton Weissenbacher, Andreas Wanninger

**Affiliations:** ^1^Department of Evolutionary Biology, University of Vienna, Vienna, Austria; ^2^Haus des Meeres, Vienna, Austria; ^3^Tiergarten Schönbrunn, Vienna, Austria

**Keywords:** Cnidaria, upside-down jellyfish, neurogenesis, myogenesis, planula, polyp, planuloid, budding

## Abstract

The scyphozoan *Cassiopea xamachana* is an emerging cnidarian model system for studying regeneration, animal-algae symbiotic relationships, and various aspects of evolutionary biology including the early emergence of animal nervous systems. *Cassiopea* has a life cycle similar to other scyphozoans, which includes the alternation between a sessile, asexual form (polyp) and a sexually reproducing stage, the medusa. The transition between the two forms is called strobilation, where the polyp releases a miniature medusa, the iconic ephyra, that subsequently develops into the adult medusa. In addition, *Cassiopea* polyps may reproduce asexually by budding off free-swimming so-called planuloid buds. While the development of planuloid buds and polyps has been studied in some detail, little is known about the ontogeny of the sexually produced planula larva. Using immunofluorescence labeling and confocal microscopy, we examined neuromuscular development during metamorphosis of the planula larva into the juvenile polyp in *C. xamachana.* For this purpose, we used tyrosinated α-tubulin-, FMRFamide- and serotonin-like immunoreactivity together with phalloidin labeling. Our results show a planula nervous system that consists of a basiectodermal neural plexus with mostly longitudinally oriented neurites. This neural meshwork is connected to sensory neurons in the superficial stratum of the ectoderm, which are exclusively localized in the aboral half of the larva. During settlement, this aborally concentrated nervous system of the planula is replaced completely by the orally concentrated nervous system of the polyp. Adult polyps show an extensive nerve net with a loose concentration around the oral disc. These findings are consistent with data from other scyphozoans and most likely constitute a conserved feature of scyphozoan discomedusae. Taken together, the data currently available suggest an aborally concentrated nervous system including sensory cells as part of the neural ground pattern of cnidarian planula larvae. The reorganization of the nervous system from anterior to posterior in planula-to-polyp metamorphosis most likely also constitutes an ancestral trait in cnidarian evolution.

## Introduction

Cnidaria comprises more than 10.000 extant species, including corals, sea anemones, and medusozoans, that predominantly inhabit marine habitats (e.g., Zhang, 2011; Appeltans et al., 2012). The phylum is widely considered the sister group to Bilateria, having separated from each other in the Precambrian over 600 million years ago (Medina et al., 2001; Hejnol et al., 2009; Dunn et al., 2014; Moroz et al., 2014; dos Reis et al., 2015). This phylogenetic position makes Cnidaria a key phylum for research into various aspects of organ system evolution, including the nervous system (Kelava et al., 2015). While poriferans and placozoans lack distinct neurons (Galliot et al., 2009), Cnidaria and Bilateria share a nervous system with complex sensory elements (Gröger and Schmid, 2001). Ctenophores also have elaborate neural features including sensory organs, but homology between ctenophore and bilaterian/cnidarian neurons is heavily debated (e.g., Moroz et al., 2014; Jékely et al., 2015; Bosch et al., 2017; Burkhardt et al., 2023).

While three germ layers are present in bilaterians, cnidarians are diploblastic with a mesoglea separating the outer ectodermal and the inner endodermal tissues. Furthermore, their gastrovascular cavity has one oral opening instead of a through-gut (Nakanishi et al., 2012). Other typical features are radially symmetric life cycle stages, multiple sets of tentacles, and the phylum-specific stinging cell type, the cnidocytes (Putnam et al., 2007). Cnidaria includes two sister lineages, Anthozoa (sea anemones, corals) and Medusozoa (Collins et al., 2006; Putnam et al., 2007). Medusozoa contains the class-level taxa Staurozoa, Hydrozoa, Scyphozoa, and Cubozoa, whose internal phylogeny is still debated (Kayal et al., 2018). Typically, medusozoans reproduce sexually in the adult medusa stage. Their ciliated planula larvae metamorphose into sessile polyps, which in turn asexually produce free-swimming medusae through strobilation (Müller and Leitz, 2002). Life cycles are, however, highly variable across Medusozoa. Hydrozoan species have evolved alternative life cycles without the medusa stage (Steele, 2002) and cubozoan taxa show a direct metamorphosis of the polyp into the medusa (Toshino et al., 2015). Scyphozoans, too, show various taxon-specific innovations, where either the planula or the polyp stage may be lost or the strobilation process (the typical process of the polyp to release ephyrae, the juvenile form of scyphozoan medusae) is reduced (Helm, 2018). Polyp stages often exhibit additional types of budding, e.g., to form colonies or produce independent individuals (Fautin, 2002).

The nervous system of cnidarians mainly consists of two nerve nets, a continuous net associated with muscle fibers and a second net which seems to have pacemaker functions (Kass-Simon and Hufnagel, 2015). In scyphomedusae, the former was described as the motor nerve net (MNN) (also giant fiber nerve net, GFNN) and the latter as diffuse nerve net (DNN). The MNN activates the swimming musculature (Zimmerman et al., 2019), while the DNN is proposed to conduct sensory information across the entire body (Satterlie and Eichinger, 2014). Cnidarian nerve nets may also contain nerve tracts accompanying muscle fibers, e.g., in the tentacles of polyps (Kass-Simon and Hufnagel, 2015). Ring nerves as loose regionalizations are known from anthozoans, hydrozoans, scyphozoans, and cubozoans. They generally circle the hypostome in polyps or the margin of the bell in medusae (Chapman, 1978; Garm et al., 2007; Marlow et al., 2009; Hufnagel and Kass-Simon, 2016; Khabibulina and Starunov, 2020). In addition to nerve nets, scypho-and cubomedusae possess rhopalia, complex sensory organs that typically contain statocysts, ocelli, and sensory hairs. They bear clusters of sensory and ganglion cells and are lacking in polyps (Nakanishi et al., 2009; Kass-Simon and Hufnagel, 2015; Rentzsch et al., 2017). Planula larvae also possess a nervous system, which is mostly aborally concentrated. It includes ectodermal (putative) sensory cells that are immunoreactive against neuropeptides such as (FM)RFamide, GLWamide, or RPamide (Nakanishi et al., 2008; Marlow et al., 2009; Piraino et al., 2011; Zang and Nakanishi, 2020). The planulae of anthozoans and the scyphozoan *Aurelia aurita* possess an apical organ with putative chemo-and/or mechanosensory functions (Chia and Koss, 1979; Nakanishi et al., 2008).

Three types of nerve cells are common in Cnidaria. Ganglion cells with basi-epithelial somata serve functions similar to interneurons. Sensory cells have an apical cilium and an elongated cell body. Both types can form synaptic contacts with muscle cells and other neurons (Rentzsch et al., 2017). Interestingly, the phylum-specific cnidocytes, one of the most complex metazoan cell types, are sometimes considered a neuronal derivative. This view is supported by their mechanosensory function and the ability of some nematocytes to form neurites and synapses with other cells (Thurm et al., 2004; Watanabe et al., 2009).

**Figure 1:**
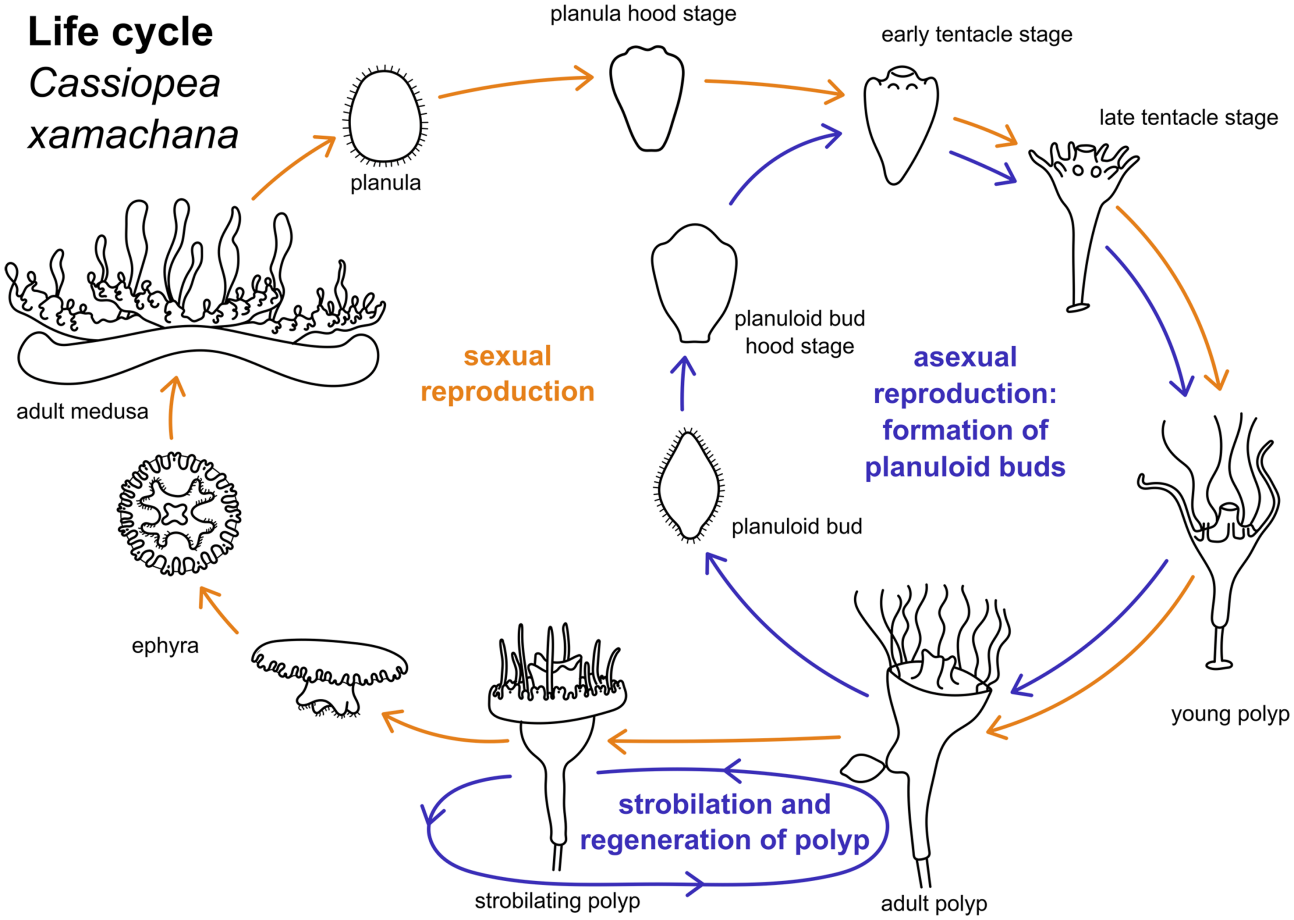
Life cylce of *Cassiopea xamachana*. Yellow arrows indicate path of sexual reproduction, blue arrows mark the asexual part of the life cycle. Adult medusae reproduce sexually to generate planula larvae. These undergo settlement and metamorphose into an adult polyp. Metamorphic stages can be separated into hood stage, early and late tentacle stage, and young polyp. Adult polyps bud off one ephyra at a time by strobilation. This ephyra develops into an adult medusa. Polyps may strobilate multiple times. Adult polyps may also bud off planuloid buds. These metamorphose into an adult polyp similar to the planula larva.

The upside down jellyfish, *Cassiopea xamachana* (Bigelow, 1900), is an emerging model system for scyphozoans (Ohdera et al., 2018). The medusae of *C. xamachana* produce planula larvae through fertilization with male sperm (Figure 1). Larvae are wrapped in a mucus envelope on the oral disc of the female medusa until they hatch and swim by ciliary beating (Hofmann et al., 1996). The anterior pole of the swimming planula attaches to the substrate and forms the future foot and stalk of the polyp. The posterior end is the oral pole which gives rise to the mouth and tentacles during metamorphosis (Neumann, 1979). Polyps can reproduce asexually by strobilation (this results in the iconic ephyra, which subsequently develops into the mature medusa) and by budding (Figure 1). In the budding process, spindle-shaped larva-like buds are formed at the lower part of the calyx. These are often referred to as “planuloid buds “(Lieshout and Martin, 1992). When the budding process is completed, the planuloid detaches and swims by ciliary activity with the aboral pole pointed forward (Martin and Chia, 1982). The free-swimming bud settles with the opposite (i.e., oral) pole and metamorphoses into a polyp, similar to the planula larva.

*Cassiopea xamachana* has received some attention for research into the interaction with endosymbiotic microalgae, Symbiodiniaceae, which are hosted in polyps, planuloids, ephyrae, and medusae (Hofmann et al., 1978, 1996; Verde and McCloskey, 1998; Ohdera et al., 2018). Recently, other topics such as regeneration (Gamero-Mora et al., 2019; Ostendarp et al., 2022; Mammone et al., 2023), the composition and function of the blue pigment present in the medusa (Phelan et al., 2006), its stinging cassiosomes (Ames et al., 2020), and neuromuscular anatomy, with focus on the medusa stage, were also targeted (Passano, 2004; Santhanakrishnan et al., 2012). The present study focuses on the neuromuscular development from the planula larva through metamorphosis into the juvenile polyp. To this end, phalloidin and immunofluorescence labeling with antibodies against FMRFamide, tyrosinated alpha-tubulin, and serotonin were used in combination with confocal microscopy. In addition, the tyrosinated tubulin- and serotonin-like immunoreactive (LIR) nervous system was investigated in planuloid buds, to fill gaps in knowledge on the neuroanatomy of this life cycle stage. The results presented herein provide a framework for a better understanding of the evolution of the complex morphological changes present in the life cycle of scyphozoans and cnidarians in general.

## Materials and methods

### Animals

Planuloid buds, metamorphosing buds, and polyps of *Cassiopea xamachana* were kindly provided by the Haus des Meeres Aqua Terra Zoo, Vienna, Austria. The planula larvae and their developmental stages were provided by Tiergarten Schönbrunn in Vienna, Austria. The polyps and their asexually produced planuloid buds and ephyrae were cultured in our lab in transparent 3 L containers with artificial sea water (Tropic Marine Inc., Germany) with a salinity of 38‰. Animals were cultured at a water temperature of 24–26°C by placing heat mats below the containers. The containers were equipped with air pumps for water circulation and bivalve shells (*Venerupis*) as settlement substrate. The animals were regularly fed with *Artemia salina* nauplii. They were exposed to a 12 h/12 h day/night cycle. The photosynthetic active radiation (PAR) light intensity under which the animals were cultured was 70μE/m2/s for the medusae and 5μE/m2/s for the polyps (Apogee Quantum Meter Modell QMSS, Ser. Nr. ELEC-1075, Apogee Instruments Inc., Logan, USA). The other developmental stages were maintained under a regular interior light regime. Ephyrae, juvenile polyps, as well as strobilating and budding polyps were taken directly from the containers and fixed the same day or one day after as described below. Polyps settling on mussel shells were carefully removed and the stalk was freed from the surrounding algae using dissecting needles.

Free-swimming planuloid buds were pipetted from the containers and kept in Petri dishes to monitor further development into young polyps. Petri dishes were equipped with bivalve shells (*Dreissena*) as a settlement substrate. The water temperature was kept between 20°C and 22°C. Under these conditions, development into a fully functioning young polyp took 3–6 days in most individuals. Developmental stages of interest were fixed as described below.

Planula larvae were obtained from one-year old medusae maintained at the Tiergarten Schönbrunn, Vienna, Austria. The planula larvae were pipetted directly from the oral disc of gravid females. Most larvae were already capable of swimming, while others were still bound together in the mucus envelope. About 300 free-swimming larvae were fixed as described below. To obtain all further developmental stages through metamorphosis into the polyp stage, planula larvae were kept in Petri dishes in the same manner as the planuloid buds.

### Fixation

The specimens were anesthetized through dropwise addition of 7.3% MgCl_2_ to the seawater until muscle contractions had fully ceased (i.e., after about 3 min.). Fixation was done in 4% paraformaldehyde in 0.1 M phosphate buffer (PB) at a pH of 7.3. The specimens were fixed at 20–23°C for 2 h, or overnight at 4°C. Fixation was followed by four washes, 10 min each, in 0.1 M PB + 0.1% NaN_3_ (pH 7.3) and the specimens were stored in the same solution at 4°C.

### Immunocytochemistry and confocal microscopy

The fixed and stored animals were first washed three times for 10 min each with PBT (0.1 M phosphate buffer + 2% Triton-X 100; pH 7.3). The planuloid buds and their developmental stages were then incubated for 1 h in 0.4 M glycine in PB to block free aldehydes, which can be unspecific binding sites for the primary or secondary antibodies. Following this, the samples were washed again three times for 10 min each with PBT. All samples were then incubated overnight in a blocking solution of 3% normal goat serum (Lifetech invitrogen, Waltham USA, PCN 5000) in PBT. Next, the specimens were incubated in primary antibodies diluted in blocking solution for 24 h at room temperature. The primary antibodies used were: (i) anti-FMRFamide (polyclonal, raised in rabbit, Biotrend, Cologne, Germany, cat. No. 1155–0100) at concentrations of 1:700–1:900, (ii) anti-tyrosinated alpha-tubulin (raised in mouse, Sigma, St. Luis USA, cat. No. 19028) at concentrations of 1:500–1:700, and (iii) anti-serotonin (raised in rabbit, Immunostar, Hudson USA, cat. No. 20080) at concentrations of 1:600–1:800. After washing the samples five times for 10 min each with PBT, the secondary antibodies were added. Goat anti mouse Alexa Fluor 488 (Invitrogen, Waltham USA, cat. No.: 100880) or goat anti rabbit Alexa Fluor 488 (cat. No.: A-11008) were used, each diluted 1:700 in PBT. For some samples, the fluorescent nucleic acid dye DAPI (Invitrogen, Waltham USA, cat. No.: D-1306) was added to the secondary antibody solution at a concentration of 1:1000. Phalloidin (Alexa Fluor 633, Invitrogen, Waltham USA, cat. No.: A12379, diluted 1:40 in PBT) was used to label filamentous actin. Specimens were incubated with secondary antibodies and/or the fluorescent dyes DAPI and phalloidin for 48 h at 4°C. Thereafter they were washed five times for 10 min each and mounted on microscope glass slides using Fluoromount-G (SouthernBiotech, Birmingham USA, cat. No.: 0100–01).

For negative controls, either the primary or the secondary antibody or both were omitted to assess antibody binding specificity and intrinsic autofluorescence of the specimens. Only the endosymbiotic microalgae show autofluorescence in the red color spectrum (633 nm). These are acquired by the planula larva via the mouth opening (Hofmann et al., 1996) and were thus present in all subsequent stages, i.e., during (larval and planuloid) metamorphosis, in the polyps, as well as in the ephyrae, medusae, and planuloid buds. All secondary antibodies revealed a non-specific signal in the aboral pole of the planuloid bud and, to a lesser degree, of the planula larvae. This area, including the putative apical organ, was therefore not included in the analyses. Non-specific signal from the secondary antibodies was also found in the acellular peduncle, below the stalk. However, this bodypart was not included in this work, as it is devoid of neuromuscular structures.

The samples were examined using a Leica SP5 confocal microscope with Leica Application Suite, Version 2.6.3.8173 software (both Leica Microsystems GmbH, Wetzlar, Germany). The confocal stacks were viewed and processed using the program ImageJ (ImageJ 1.46r software, National Institutes of Health, Bethesda, Maryland) (Schneider et al., 2012).

### Light microscopy and image processing

Live animals were examined and recorded using a Nikon SMZ 25 stereo microscope with the imaging software NIS-Elements (BR 5.01.00 64-bit.Ink; Nikon Corporation, Tokyo, Japan). Further image processing, annotations, and figure plate assembly was done in GIMP (Gnu Image Manipulation Program, Version 1.10.24, GNU general public licence) and Inkscape (Version 1.2, GNU general public licence). Schematic illustrations were also created in GIMP and Inkscape.

## Results

### *Cassiopea xamachana* life cycle

**Figure 2:**
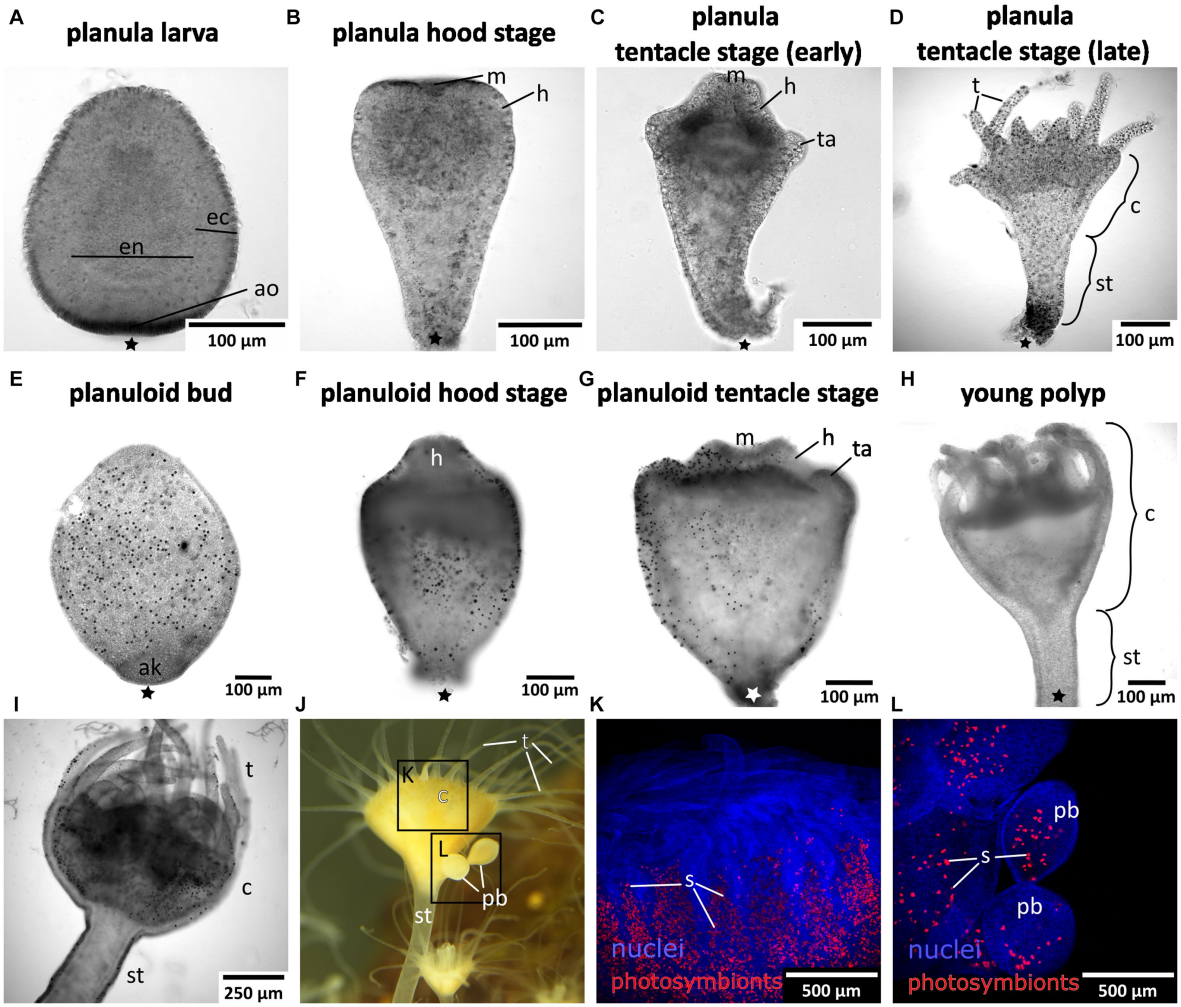
Light and fluorescence micrographs of the life cylce stages investigated herein. Asterisks mark the aboral pole. **(A)** Early planula larva with distinct ectoderm (ec) and endoderm (en). **(B)** Planula hood stage with mouth (m) and forming hypostome (h). **(C)** Planula tentacle stage with tentacle anlagen (ta). **(D)** Late tentacle stage that emerged from a sexually produced planula larva with developing tentacles (t), calyx (c), and stalk (st). **(E)** Young asexually produced planuloid bud with the aboral knob (ak) at the aboral pole. Black dots are photosymbionts. **(F)** Planuloid hood stage with the hypostome forming. **(G)** Planuloid tentacle stage with mouth and tentacle anlagen. **(H)** Young polyp that developed from an asexually produced planuloid bud. **(I)** Adult polyp with dinstinct stalk, calyx, and fully developed tentacles. **(J)** Adult polyp with two planuloid buds (pb). **(K)** Calyx of an adult polyp with nuclei in blue and the autofluorescing photosymbionts (s) in red. **(L)** Planuloid buds attached to the calyx, nuclei in blue and photosymbionts in red.

Mature medusae reproduce sexually, resulting in free-swimming planula larvae that emerge from the oral disc of the female (Figures 1, 2A). In the present study, developmental times varied considerably, with some individuals commencing settlement and metamorphosis after 4 days, while the majority underwent metamorphosis only after approximately 2 weeks. Accordingly, settlement may start as early as in the free-swimming stage, but may take until the hood or early tentacle stage. In our experiments, most individuals started settlement in the hood stage (see Figure 1 and below). At 20–22°C, metamorphosis took about 3 days from settlement until formation of the young polyp. First, the elongated animal changes to a pear-like shape with a slimmer aboral end. The hypostome forms at the oral pole and expands in oral direction with the mouth opening on the oral pole. This is referred to as the hood stage (Figures 1, 2B). Below the hypostome, the tentacle anlagen develop (Figure 2C). This is referred to as early tentacle stage (Figures 1, 2C,D). The stalk elongates, and calyx and stalk are still confluent. Until the polyp stage, additional tentacles are successively added (late tentacle stage) (Figures 1, 2D). Stalk and calyx develop further, until a distinct cup-like calyx and a slim, straight stalk are established (Figure 2H). A number of long tentacles have formed by then. This stage is referred to as young polyp herein (Figure 1). Further growth and development result in the adult polyp stage, which is capable of asexual reproduction by budding or strobilation (Figures 1, 2I–L). At 20-22°C, development of the planuloid buds (Figure 2E) into the polyp takes 3-6 days after detachment. Although larvae and buds are both roundish and move by ciliary activity, there are gross morphological differences, especially in size and shape (Figures 2E–G). Planuloid buds are larger and have a more pear-like shape with a distinct aboral knob that is less ciliated. Moreover, they already bear the endosymbiotic zooxanthellae (Figure 2L). In planuloid buds, the same metamorphic stages as for planulae can be observed from the swimming stage onwards. Asexual reproduction of polyps by strobilation and formation of ephyrae is followed by development into the adult medusa (Figure 1). After strobilation the polyp regenerates and is again capable of budding and strobilation.

### Planula myogenesis

**Figure 3:**
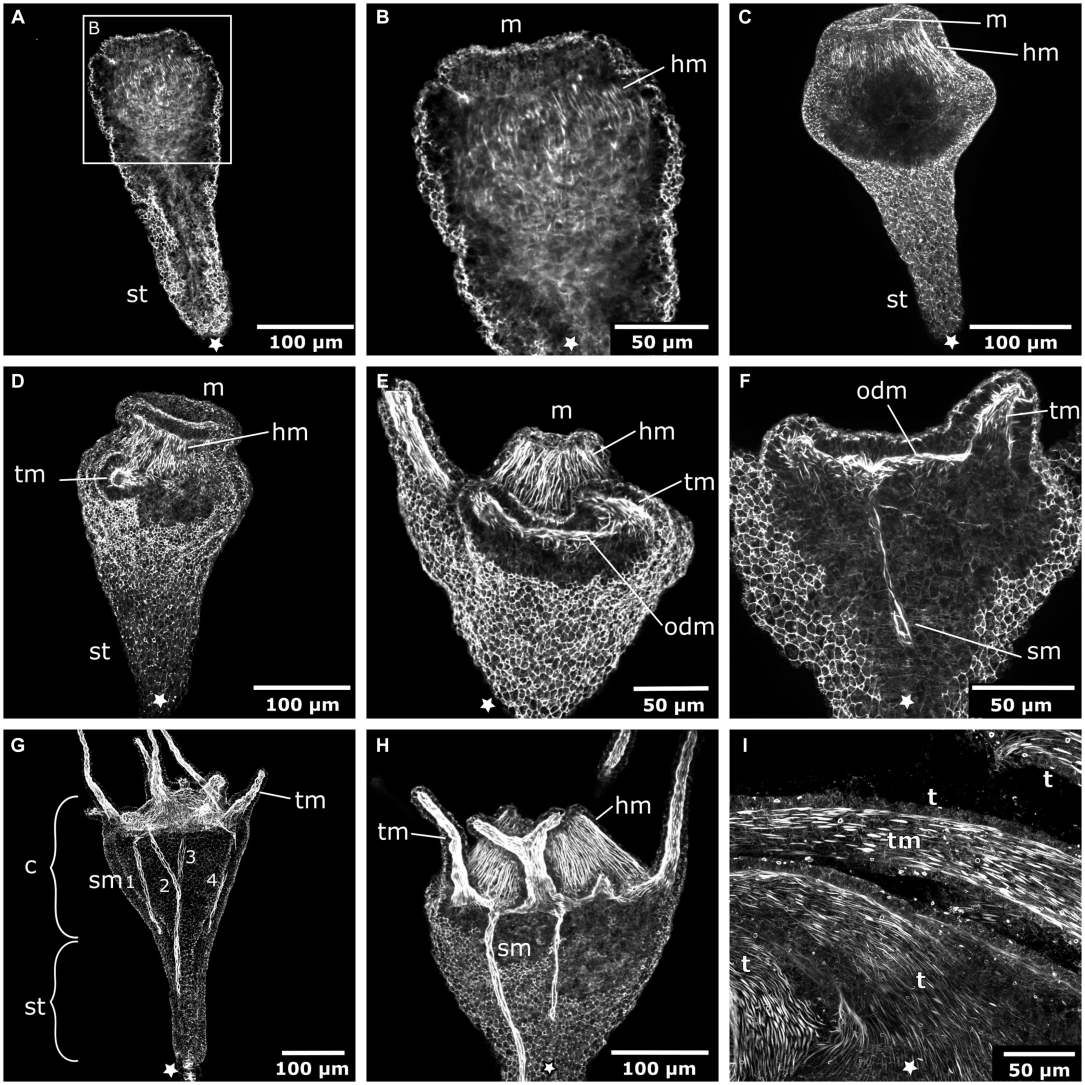
Myogenesis during *Cassiopea xamachana* metamorphosis. All panels show images of actin stainings with phalloidin. Asterisks mark the aboral pole. **(A)** Planula larva during settlement and early stalk (st) formation. **(B)** Detail of the oral (i.e., posterior) region with the mouth (m) and hypostome at the onset of hypostome muscle (hm) formation. **(C)** Hypostome musculature is elongated and the mouth opening is present. **(D)** Hypostome muscles grow in distal direction and ectodermal tentacle muscles (tm) surround each tentacle. **(E)** The tentacle (t) musculature interconnects at the margin of the oral disc and forms the oral disc muscle (odm). **(F)** From the oral disc muscle, four muscle bands grow in aboral direction and form the four septal muscles (sm). **(G)** A young polyp with the four septal muscles forming. The cup-like calyx (c) is distinct from the stalk. **(H)** Detail of the calyx with hypostome muscles, tentacle muscles, and septal muscles. **(I)** Detail of the tentacle muscles that line the entire tentacle of the adult polyp.

The early planula larva is devoid of muscular elements. Mostly concurrent with settlement, the larval oral pole undergoes visible changes. The hypostome expands orally and longitudinal muscle fibers are established in a radial pattern around the future mouth (Figures 3A–C). The muscle fibers elongate and the mouth protrudes in oral direction. The oral disc is located aborally from the hypostomal muscle fibers. At the oral disc, hypostomal muscle fibers grow in distal direction toward the tentacle anlagen (Figure 3D). As the polyp grows, the number of tentacles increases. The tentacles are lined with basiepidermal longitudinal muscle fibers from their base to the distal pole (Figures 3E–I). Muscles from each tentacle base connect with each other circumferentially around the oral disc, forming the circular oral disc muscle (Figure 3E). In the young polyp stage, muscle fibers from the oral disc muscle project in aboral direction. At four sites, prominent muscle bands elongate toward the base of the stalk, giving rise to the four septal muscles (Figures 3F–H).

### Planula neurogenesis

**Figure 4:**
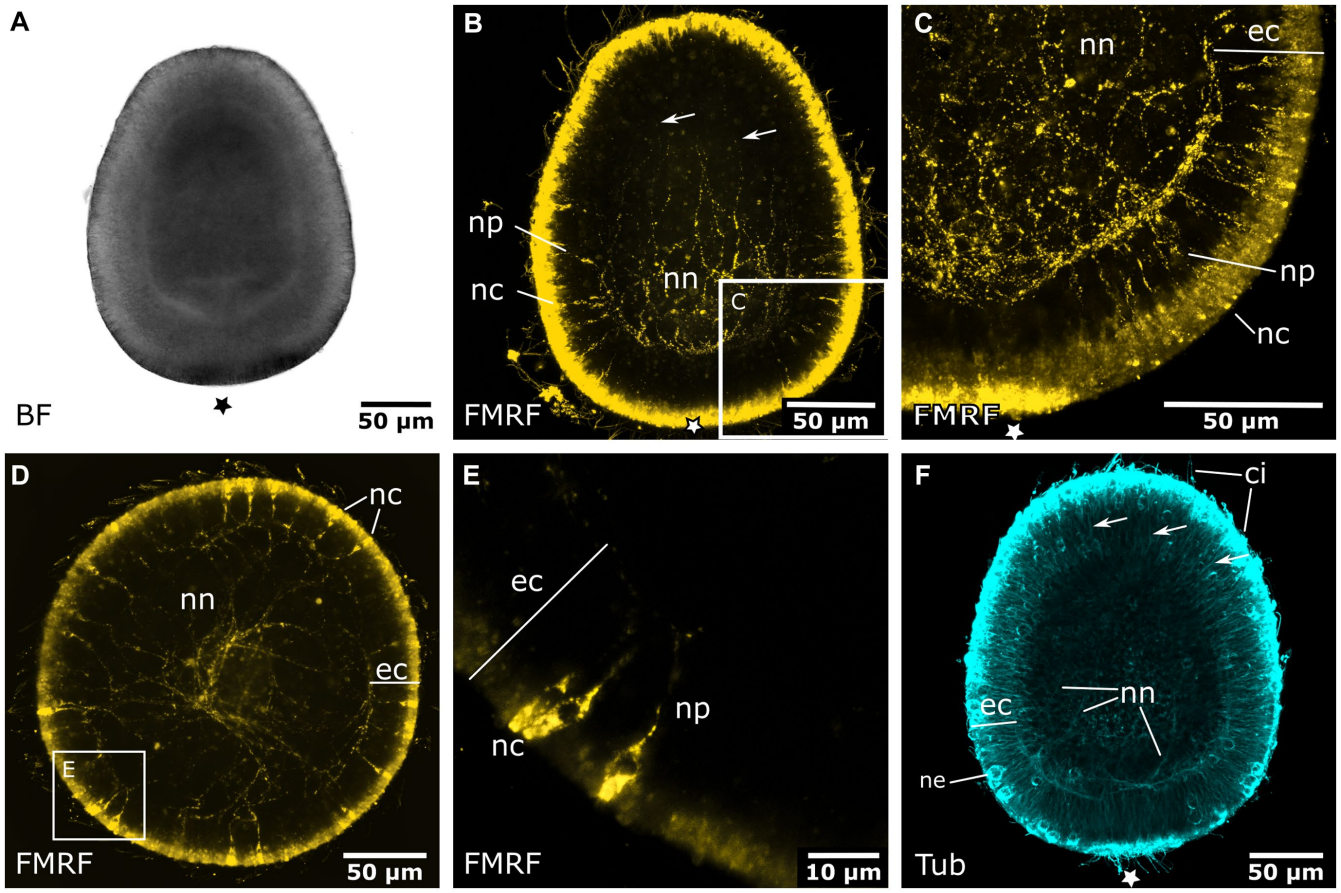
Nervous system of the early and late planula larva. Asterisks mark the aboral pole. **(A)** Light micrograph of an early planula larva. **(B)** FMRFamide-LIR nervous system of the early larva. Neural processes (np) of ectodermal neural cell bodies (nc) form a basiepidermal nerve net (nn). Arrows indicate neurite endings in the oral half. **(C)** Detail of **(B)**, neural cell bodies in the superficial stratum of the ectoderm (ec) are connected to the nerve net at the base of the ectoderm by neurites (np). **(D)** 8-day old free-swimming planula larva. Oral-aboral view of the midbody region. **(E)** Same specimen as in **(D)**. Detail of three neural cell bodies with processes directed basiectodermally. **(F)** Tyrosinated tubulin antibody staining of an early planula larva showing fibers of the nerve net (nn) and fibers crossing the ectoderm in proximal-distal direction (arrows). Nematocytes (ne) and cilia (ci) also show tyrosinated tubulin-LIR.

Early planula larvae (Figure 4A) show FMRFamide-like immunoreactive (FMRFa-LIR) ectodermal neurons that are connected to a basiectodermal neural plexus (Figures 4B,C). The neurons are located in the superficial stratum of the ectoderm and are evenly distributed over the aboral half of the spherical larva (Figure 4E). After a transitional zone in the middle of the larva, neurons become fewer in number and are absent from the oral half. Due to the non-specific reaction of the secondary antibodies on the apical (i.e., aboral) pole, the presence or absence of neurons in this region could not be assessed. The neurites of each neuron face inwards toward the base of the ectoderm. Here, they connect to the neural plexus, whose neurite bundles are mostly oriented longitudinally. The FMRFa-LIR neurites project into the oral half, spanning over more than three quarters of the larva, with a decreasing signal intensity in oral direction (Figure 4B). Neurites in the ectodermal neural plexus also show tyrosinated tubulin-like immunoreactivity (tyr. tubulin-LIR), but at a low intensity. Except for the neural plexus in the basal layers of the aboral ectoderm, the whole planula shows thin tyr. tubulin-LIR fibers spanning throughout the ectoderm (Figure 4F).

**Figure 5:**
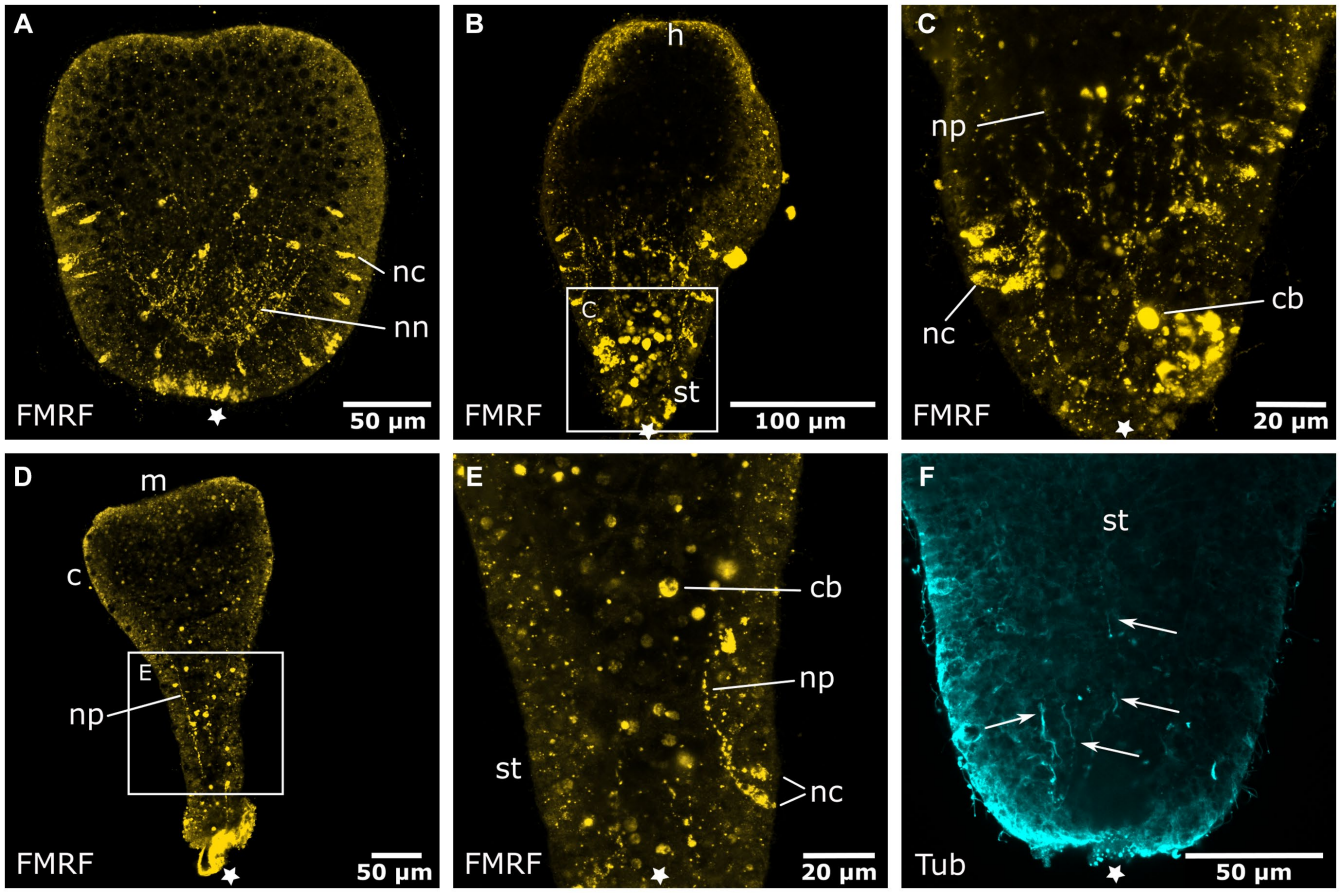
Degeneration of the aboral nervous system in the planula larva. Asterisks mark the aboral pole. **(A)** The hood stage shows a decreased number of neural cell bodies (nc) in the ectoderm and neurites of the basiepidermal nerve net (nn). The long neurites in the oral half have been reduced. **(B)** Larva during settlement and formation of the stalk (st) and hypostome (h). **(C)** Detail of **(B)**, neurons and neurites (np) disintegrate and FMRFamide-LIR bodies (cb) appear in the endoderm. **(D)** Settlement is completed, a few remaining neurites of the aboral nervous system are still present. The calyx (c) and mouth (m) are formed but lack a neuronal innveration. **(E)** Detail of **(D)** with degenerating neurons and neurites in the stalk. **(F)** Detail of the stalk with anti-tyrosinated tubulin staining shows solitary thin fibers (indicated by arrows) that form at the base of the stalk and grow in oral direction.

The larval aboral nervous system starts to degenerate during settlement in the hood stage. This process is accompanied by external visible changes, such as the shortening of the larva, the subsequent formation of the mouth, and the elongation of the stalk. The long neurites of the plexus, which branch across the oral half, seem to disintegrate first and the number of FMRFa-LIR neurons appears to decrease (Figure 5A). Subsequently, neurons without the regular oval shape are present and individual nerve fibers show less prominent and more scattered immunoreactive signal (Figures 5B,C). Such changes are common for disintegrating neural elements in marine invertebrate larvae, although exprimental data such as TUNEL assays would be required for a definite proof of loss of these structures due to apoptosis. Once the mouth opening is fully formed and the stalk elongated, single nerve cells or fibers are still visible, but are lost entirely in the tentacle stage (Figures 5D,E). Inside the endoderm, FMRFa-LIR bodies of different sizes and without visible neurites accumulate (Figures 5B–D). These might be fragments of apoptotic neurons (Nakanishi et al., 2008). In the late hood stage, tyr. tubulin-LIR neurites are present in the ectoderm near the aboral pole (Figure 5F).

**Figure 6:**
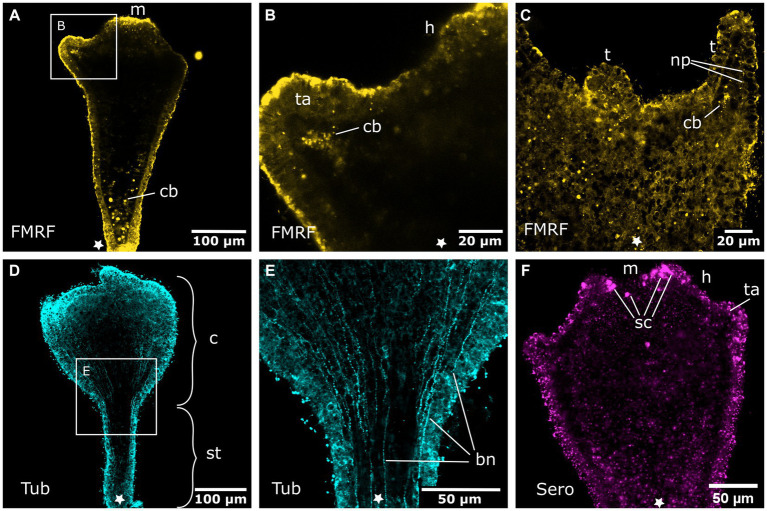
Nervous system in sexually produced tentacle stages. Asterisks indicate the aboral side. **(A)** Tentacle stage larva with fully formed mouth (m). It lacks an FMRFamide-LIR aboral nervous system in the ectoderm. Only FMRFamide-LIR cell bodies (cb) in the endoderm are present. **(B)** Detail of **(A)** with tentacle anlagen (ta) emerging aborally to the hypostome (h). They show endodermal FMRFamide-LIR cell bodies (cb). **(C)** Growing tentacles (t) with first sparse FMRFamide-LIR neural processes (np) in the ectoderm. **(D)** Tentacle stage larva showing tyrosinated tubulin-LIR longitudinal neurites that extend along the entire body, from the base of the stalk (st) to the calyx (c) and tentacles. **(E)** Detail of **(D)** with longitudinal solitary body neurites (bn). **(F)** Serotonin-LIR cells (sc) in the vicinity of the mouth opening.

**Figure 7:**
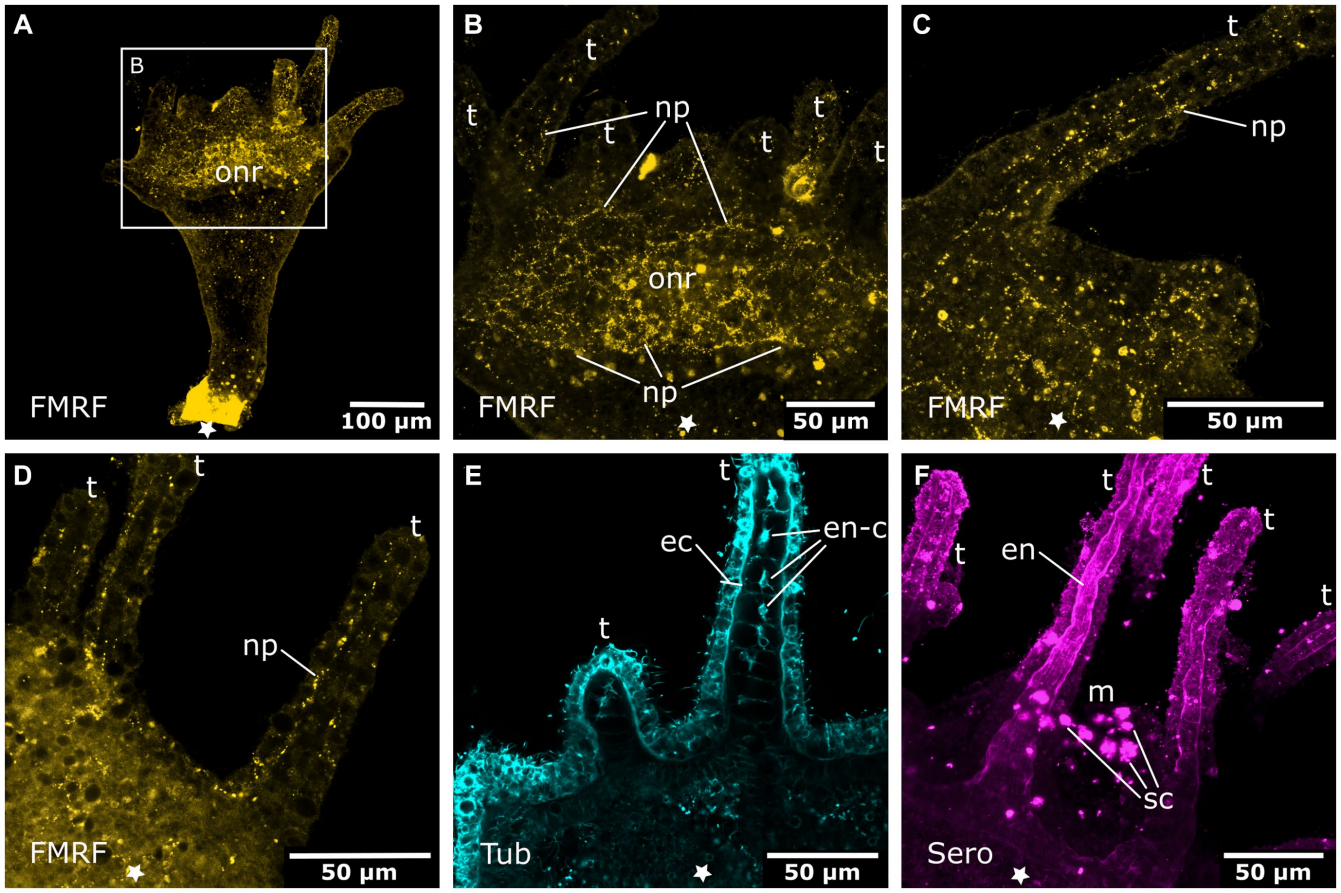
Oral nervous system of sexually produced polyps. Asterisks indicate the aboral side. **(A)** Developing polyp at the late tentacle stage with an oral nervous system that is concentrated around the oral disc in a loose oral nerve ring (onr). **(B)** Detail of **(A)**, the oral nerve ring is situated below the tentacles (t) and consists of numerous FMRFamide-LIR neural processes (np). **(C)** Z-projection through a tentacle that shows a meshwork of neurites (np) along the entire tentacle. **(D)** The median layer of the tentacles shows their position in the basal ectoderm. **(E)** Developing tentacles also show tyrosinated tubulin-LIR endodermal cells (en-c). The intense staining of the ectoderm (ec) is due to immunoreactive cilia and nematocytes. **(F)** Serotonin-LIR cells (sc) around the mouth (m) opening and serotonin-LIR in the tentacle endoderm (en).

**Figure 8:**
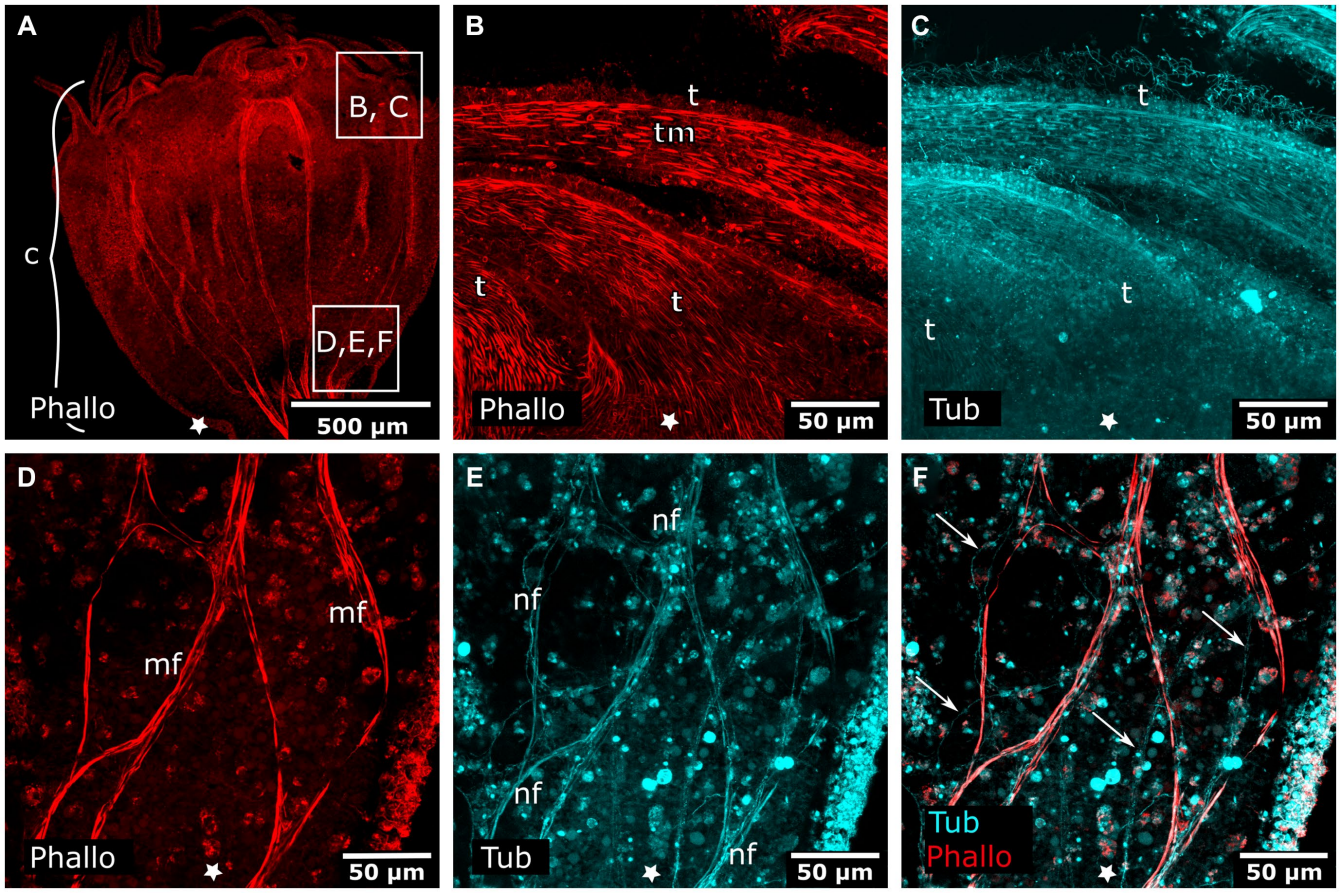
Combined phalloidin and tyrosinated tubulin staining in adult polyps. Asterisks indicate the aboral side. **(A)** Calyx (c) of an adult polyp. **(B)** Detail of tentacles (t) with tentacle musculature (tm). **(C)** Same sections as in **(B)** labeled with anti-tyrosinated tubulin show muscles, nematocytes, and cilia. **(D)** Detail of a planuloid bud-forming site at the lower calyx area. Phalloidin staining showing branched muscle fibers (mf). **(E)** Same section as in **(D)**, tyrosinated tubulin-LIR fibers (nf) are found at the same position as muscle fibers. **(F)** Same section as in **(D,E)**, double labeling with phalloidin and anti-tyrosinated tubulin. Arrows mark neural fibers that do not strictly follow a muscle fiber.

In the tentacle stage, the aboral nervous system is mostly degenerated and the tentacle anlagen start to develop. Few FMRFa-LIR bodies (apoptotic fragments?) remain in the aboral endoderm. In the region of the mouth and the oral disc no FMRFa-LIR is found. The tentacle anlagen show endodermal cells with FMRFa-LIR components (Figures 6A,B). Once this endodermal FMRFa-LIR disintegrates, scattered FMRFa-LIR neurites can be detected within the developing tentacle knobs (Figure 6C). No clear tyr. tubulin-LIR nervous system components can be determined in the anlagen of the tentacles and the oral disc. This is probably due to a high amount of immunoreactive nematocytes, cilia, and muscle fibers in the ectoderm, which obscure the considerably less intense signal of the neural elements (Figure 6D). The problem persists through further developmental stages until the adult polyp has developed (Figures 7E, 8A–C). Apart from nematocytes and cilia, the tyr. tubulin-LIR labeling in the tentacles of adult polyps also stains the musculature and thus highly resembles the phalloidin signal that visualizes the tentacle muscles (*cf.* Figures 8B,C). Accordingly, distinct tyr. tubulin-LIR neurites could not be identified in the tentacles of adult polyps. This is different for other body regions of the polyp, e.g., the bud-forming sites, where tyr. tubulin-LIR neural components that lack phalloidin-positive signal can readily be distinguished from muscle fibers (Figures 8D–F).

The stalk and calyx do show tyr. tubulin-LIR neurites in the superficial layers of the ectoderm. Solitary, thin, longitudinal fibers stretch all over the stalk and calyx, reaching up to the tentacle anlagen (Figures 6D,E). In the tentacle stage, the first serotonin-like immunoreactive (serotonin-LIR) cells appear around the margin of the mouth opening (Figure 6F).

In the late tentacle stage, a net of FMRFa-LIR fibers forms that spans the oral disc and hypostome (Figures 7A,B). In this and further developmental stages until the adult polyp, FMRFa-LIR neurites are present, but immunoreactive cell bodies are not detected. The FMRFa-LIR in the tentacles shows a branching nerve net that surrounds the whole tentacle ectoderm (Figures 7C,D). The tentacle endoderm consists of a single row of large, vaculous, disc-shaped cells that are stacked upon each other (discoidal endodermal cells). During tentacle growth, these cells show tyr. tubulin-LIR, which largely disappears in the young polyp stage (Figure 7E). In addition, the developing tentacle endoderm shows serotonin-LIR. Around the mouth opening and in the gastrovascular cavity, large serotonin-LIR cells are present (Figure 7F). The whole stalk and calyx ectoderm is now covered with evenly spaced tyr. tubulin-LIR fibers that extend in longitudinal direction (solitary body neurites).

**Figure 9:**
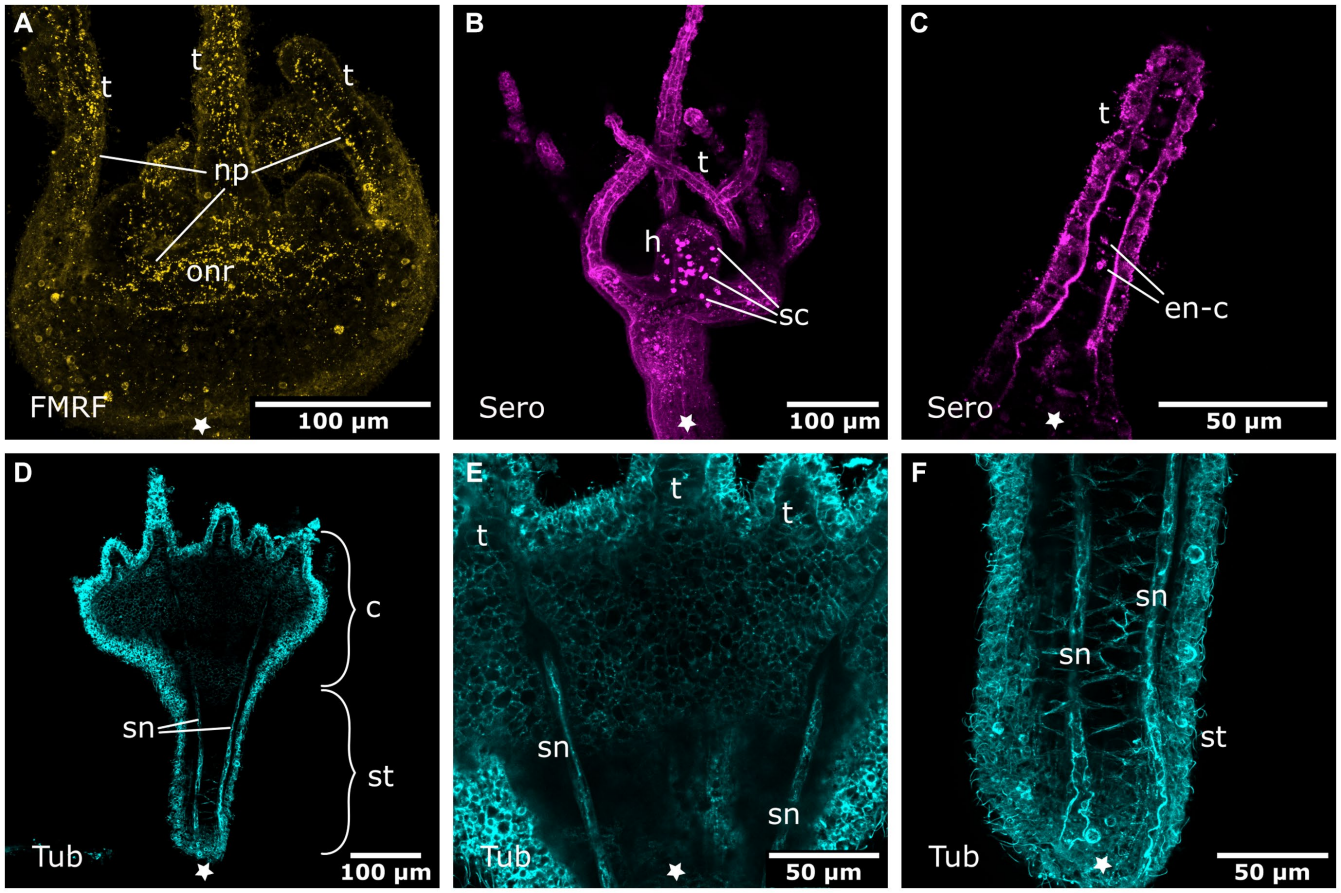
Nervous system in young polyps. Asterisks indicate the aboral side. **(A)** Oral nervous system concentrated in an oral nerve ring (onr). Tentacles (t) and hypostome are innervated by numerous neuronal processes (np). **(B)** Serotonin-LIR cells (sc) in the hypostome (h). **(C)** Discoidal serotonin-LIR endodermal cells (en-c) in the tentacles. **(D)** Tubulin-LIR septal neurites (sn) accompanying the septal muscle bands from the base of the stalk (st) through the calyx (c) to the base of the tentacles. **(E)** Detail of the calyx from **(D)** with the septal neurites terminating below the tentacles. **(F)** Detail of the stalk from **(D)** showing the septal neurites near the aboral pole.

The FMRFa-LIR oral nerve net of young polyps spans the hypostome and oral disc. Its highest condensation is the circumhypostomal nerve ring around the margin of the oral disc (Figure 9A). The tentacle ectoderm is lined with thin, branched FMRFa-LIR nerve fibers (Figure 9A). Besides the longitudinal fibers in the stalk and calyx ectoderm, new tyr. tubulin-LIR nerve fibers appear between the late tentacle stage and the young polyp stage. Alongside the four septal muscle bands, prominent septal neurites are found (Figures 9D–F). They accompany the septal muscle bands from the foot through the stalk until the base of the tentacles. Every muscle band is accompanied by a few tyr. tubulin-LIR neurites with several branches (Figures 9D–F).

Additional serotonin-LIR structures appear in the young polyp stage. The serotonin-LIR cells around the mouth increase in number. The endoderm bears a regularly dispersed arrangement of serotonin-LIR cells along the whole hypostome (Figure 9B). The fully developed tentacles also contain serotonin-LIR endodermal cells (Figure 9C). The discoidal endodermal cells of the tentacles show extranuclear, spot-like serotonin-LIR in their somata. This is only observed in fully formed tentacles, from young to adult polyp stages. All FMRFa-LIR, tyr. tubulin-LIR, serotonin-LIR neural structures, as well as the musculature, are present and are further elaborated until polyp adulthood. These findings are identical in the metamorphosing planuloid buds and planula larvae, respectively.

### Planuloid bud development

**Figure 10:**
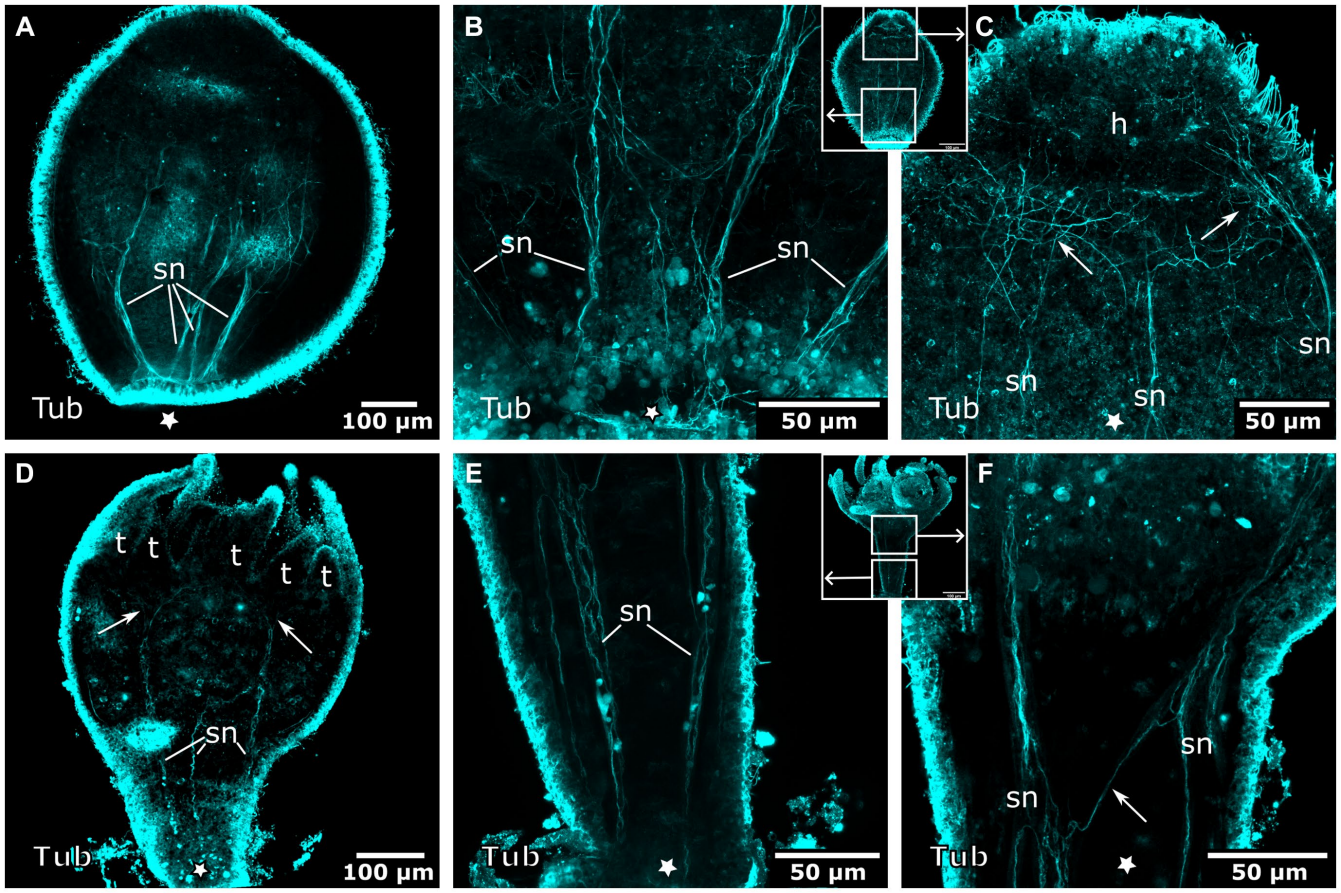
Tyrosinated tubulin-LIR nervous system during planuloid bud development. Asterisks indicate the aboral side. **(A)** Early planuloid bud with four strands of tubulin-LIR fibers. The septal neurites (sn) are already present. **(B)** Hood stage planuloid, detail of the aboral region, early formation of the septal neurites. **(C)** Hood stage planuloid, detail of the oral region, the septal neurites form numerous arborisations at the site of the developing hypostome (h) (arrows). **(D)** Metamorphosing planuloid bud during tentacle (t) formation. The septal neurites disperse near the tentacle bases (arrows). **(E)** Detail of the septal neurites in the stalk of a young polyp derived from a planuloid bud. **(F)** Detail of the septal neurites in the calyx-stalk-junction of the same polyp as in **(E)**. An arrow marks the connection between two separate strands of septal neurites.

In the budding polyp, one of the septal muscle bands branches and enters the area of the developing planuloid bud (*cf.* Khabibulina and Starunov, 2019). Thus, at detachment, buds already possess 4 septal muscles at their aboral (apical) pole. In the early detached planuloid buds, a prominent system of tyr. tubulin-LIR nerve fibers runs alongside the 4 muscle bands (Figure 10A). The neurites start at the aboral pole and grow in oral direction, where they branch into several fine fibers (Figure 10C). This neuromuscular system is present from the early planuloid stage until the adult polyp stage. However, it undergoes various changes during the growth of the planuloid to the polyp (Figures 10B–F). The tyr. tubulin-LIR neurites form interconnections, particularly at the stalk-calyx intersection. The neuronal projections arborize and terminate at the tentacle bases (Figures 10D–F). The neurites of the oral nerve ring lack tyr. tublin-like immunoreactivity.

**Figure 11:**
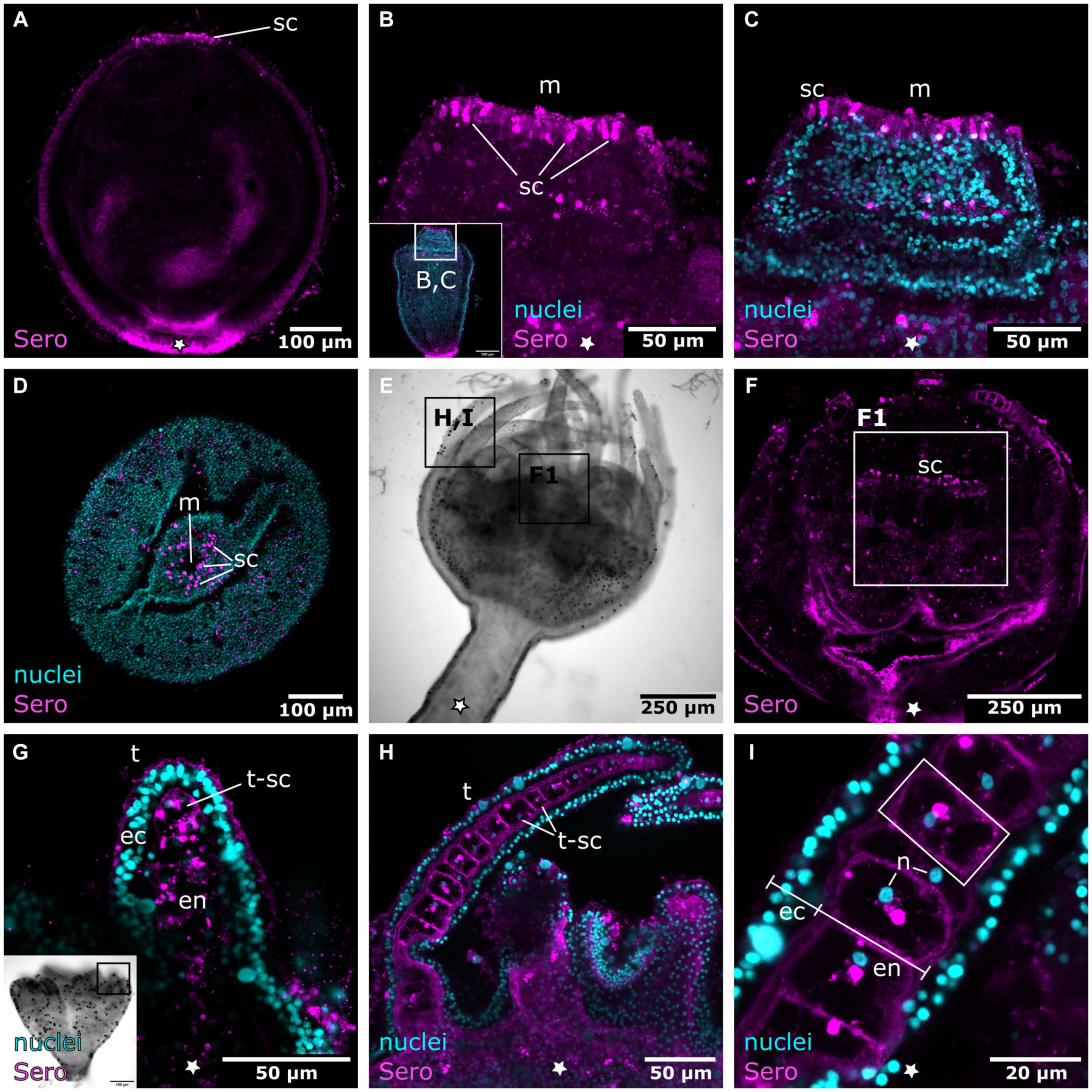
Serotonin-LIR cells in the metamorphosing planuloid. Asterisks indicate the aboral side. **(A)** Young planuloid buds show serotonin-LIR cells (sc) where the mouth will form. **(B)** Serotonin-LIR cells in the vicinity of the mouth opening (m) in the hood stage. **(C)** Same section as in **(B)**, but with nuclei (blue) and anti-serotonin (pink) labeling. **(D)** Planuloid during settlement, seen from an oral perspective. Serotonin-LIR cells are present around the mouth. **(E)** Light micrograph of a polyp. **(F)** Calyx of a polyp. F1 shows the hypostome and the serotonin-LIR cells around the mouth of a young polyp. **(G)** Emerging tentacles (t) in a metamorphosing planuloid bud. The endoderm (en) shows serotonin-LIR tentacle cells (t-sc), whereas the ectoderm (ec) shows no serotonin-LIR. **(H)** Tentacle from a young polyp with serotonin-LIR endodermal tentacle cells. **(I)** Detail of **(H)**. The endoderm consists of single, highly vacuolated, discoidal cells stacked onto each other (frame highlights a single endodermal cell). The nucleus (n) is distinct from the serotonin-LIR cell soma.

**Figure 12:**
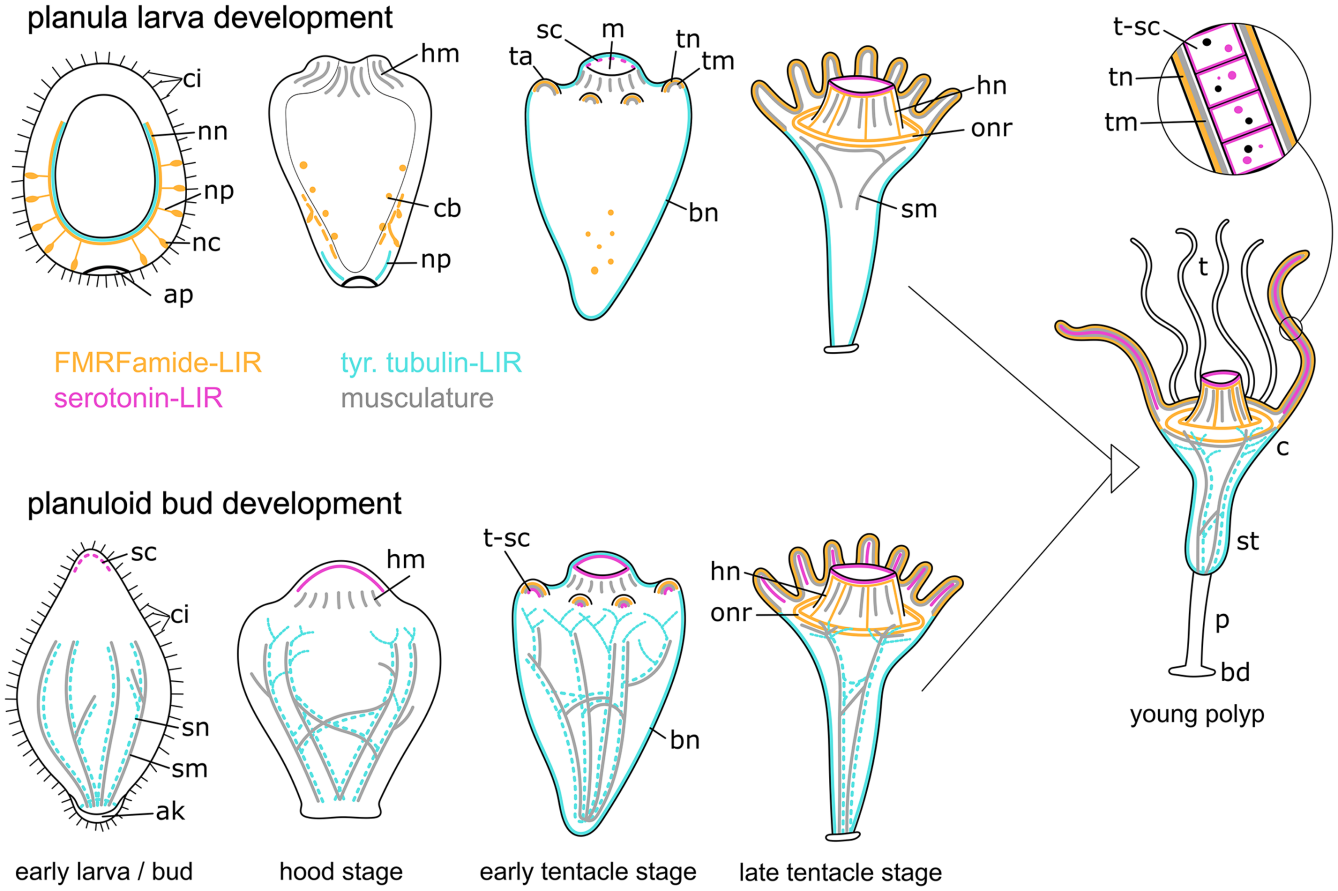
Summary of myogenesis and neurogenesis in the planula larva (upper images) and the planuloid bud (lower images) through metamorphosis and the young polyp stage. The aboral pole is directed downwards. Yellow structures represent the FMRFamide-LIR nervous system, cyan marks the tubulin-LIR nervous system, pink shows the serotonin-LIR cells, and gray depicts the musculature. Differences include: (i) the presence of the aboral nervous system in planula larvae but not planuloid buds, (ii) the presence of septal muscles (sm) as well as septal neurites (sn) in planuloid buds but not planula larvae, and (iii) the stage when serotonin-LIR cells (sc) emerge. The schematic representation also includes the results from myogenesis and FMRFamide-LIR during planuloid bud development as described in Khabibulina and Starunov (2019, 2020). Aboral knob (ak), apical pole (ap), FMRF-LIR cell bodies (cb), basal disc (bd), body neurites (bn), calyx (c), cilia (ci), hypostome muscles (hm), hypostome neurites (hn), mouth (m), nerve net (nn), neural processes (np), neural cell bodies (nc), oral nerve ring (onr), peduncle (p), serotonin-LIR cells (sc), septal muscles (sm), septal neurites (sn), stalk (st), tentacle anlagen (ta), tentacles (t), tentacle muscles (tm), tentacle neurites (tn), tentacle serotonin-LIR cells (t-sc).

Early planuloid buds show several dispersed serotonin-LIR cells at the oral pole (Figure 11A). In the hood stage, a ring of dispersed serotonin-LIR cells around the mouth opening is visible (Figures 11B–D). In the following developmental stages, such cells are found in the anterior region of the gastrovascular cavity in close proximity to the mouth, as described for the planula larva (Figure 11E,F). In planuloid buds, the tentacle anlagen already show distinct serotonin-LIR endodermal cells (Figure 11G). From the tentacle stage onwards, every tentacle endodermal cell shows extranuclear, spot-like serotonin-LIR but none of these cells exhibit neuronal processes (Figures 11E,H,I). During planula metamorphosis this condition is not present before the young polyp stage. From the young polyp stage onwards, similar results were retrieved for planuloid buds and planula larvae (Figure 12). All FMRFa-LIR, tyr. tubulin-LIR, and serotonin-LIR neural structures are present and are further elaborated until polyp adulthood.

## Discussion

### Comparison of neuromuscular remodeling during *Cassiopea xamachana* planula and planuloid bud metamorphosis

Metamorphic remodeling of the muscular architecture differs considerably between the two reproductive pathways (Figure 12). The *Cassiopea xamachana* planuloid buds inherit parts of the septal muscle from the mother organism. These muscles reorganize to form four septal muscles in the planuloid bud, which are present from the free-swimming stage onwards (Khabibulina and Starunov, 2019). This is consistent with our data on tyr. tubulin-LIR neurogenesis in planuloids. Septal neurites are also present from the free-swimming stage onwards in the planuloid bud. They already include connections to each other and ramifications in the oral region. During planula metamorphosis, the septal muscle bands as well as the septal neurites are fully developed from the young polyp stage onwards. They reach from the oral disc to the base of the stalk. There are no notable differences to the musculature of young polyps derived from either planuloid buds or planula larvae. Except for the septal muscle bands, which must form *de novo* in the planulae, all musculature present in the polyps develops at the same developmental stage and in the same manner in both developmental trajectories. These findings suggest that similar morphogenetic and molecular pathways underlie myogenesis in sexually and asexually produced offspring of *C. xamachana*.

No contribution of the FMRFa-LIR nervous system from the mother organism to the planuloid bud could be observed. Instead, all FMRFa-LIR elements of the planuloid buds seem to form *de novo* (Khabibulina and Starunov, 2020). Except for the septal neurites, FMRFa-LIR and tyr. tubulin-LIR neurogenesis are very similar in metamorphosing *C. xamachana* planulae and planuloid buds (Figure 12).

The serotonin-LIR cells from planula larvae and planuloid buds show the same results in the young polyp stage and onwards. However, the presence of serotonin-LIR cells on the oral side of the freshly detached bud suggests that some serotonin-LIR cells are also inherited from the mother organism during asexual reproduction.

### Planula myogenesis

The early *Cassiopea xamachana* planula larva is devoid of musculature and its rotating movement through the water column is mediated by cilia (Martin and Chia, 1982). Some cnidarian planulae, however, do show functional muscles, e.g., the benthic larva of the hydroid *Clava multicornis* (Forsskål, 1775), which moves by lateral bending (Piraino et al., 2011). The ectoderm of *C. xamachana* and other cnidarian planulae consists mostly of epithelio-muscular (supportive) cells (Martin and Chia, 1982; Kraus et al., 2022). Body deformations in the larva-to-polyp transition are found to be driven by muscular hydraulics in the sea anemone *Nematostella vectensis* Stephenson, 1935 (Stokkermans et al., 2022). The *C. xamachana* polyp musculature consists of four distinct groups, the septal muscles, tentacle musculature, hypostome muscles, and the circular oral disc muscle (Khabibulina and Starunov, 2019), which is consistent with our findings. The polyp musculature from *C. xamachana* is also consistent with that of *Aurelia aurita* (Linnaeus, 1758) and the general scyphopolyp body plan (Chia et al., 1984; Helm et al., 2015; Sukhoputova et al., 2019). Accordingly, this is a presumably conserved body plan of Discomedusae (the two scyphozoan clades Semaeostomeae and Rhizostomeae). There are, however, no data available for the third scyphozoan clade, the Coronatae.

### Planula neurogenesis

Most cnidarian planula larvae show an aboral (anterior) concentration of sensory neurons that may play an important role during metamorphosis (Katsukura et al., 2003; Nakanishi et al., 2008). Although earlier electron microscopy studies failed to detect neural elements (Martin and Chia, 1982), we found numerous aborally distributed putative sensory neurons in the *Cassiopea xamachana* planula larvae which degenerate during metamorphosis (Figure 12). In contrast, the orally concentrated nervous system of the polyp develops only at later stages, i.e., after settlement. The FMRFa-LIR neurons in the larval ectoderm are connected to an aborally concentrated nerve net, located at the base of the ectoderm. Due to their shape and position, these neurons likely have sensory functions, as has been described from other cnidarian planulae (Martin and Thomas, 1981; Nakanishi et al., 2008). Within Scyphozoa, the rhizostome *C. xamachana* shows a similar neural architecture as the semaeostomean planula of *Aurelia aurita.* The latter also possesses FMRFa-LIR sensory lateral neurons, which are associated with an aborally concentrated neural plexus at the base of the ectoderm (Nakanishi et al., 2008). *Aurelia aurita* is the only medusozoan for which an apical organ has been described. Furthermore, homology to the apical organ of anthozoans has been proposed (Nakanishi et al., 2008). Due to a non-specific reaction of the secondary antibodies directly on the apical pole, where the apical organ would be expected, no apical organ cells could be unequivocally identified in *C. xamachana*. Based on the results of Semaeostomeae and Rhizostomeae, neurons restricted to the aboral half of the planula seem to be a conserved feature for Discomedusae (i.e., Semaeostomeae + Rhizostomeae). In order to unambiguously assess the groundplan of Scyphozoa, data on the third scyphozoan clade, Coronatae, would be required.

Hydrozoan planulae also show a nervous system with ectodermal neurons, but they are not restricted to the aboral half as in Scyphozoa. In Scyphozoa as well as Hydrozoa the neurites from the neural plexus extend in longitudinal direction. Planulae of the hydrozoans *Pennaria tiarella, Hydractina echinata, Clava multicornis,* and *Gonothyraea loveni* show not only sensory neurons in the ectoderm, but also a neural plexus in the mesoglea that contains ganglionic cells. The ganglionic cells are distributed over the whole body axis, but with a high degree of aboral (anterior) concentration (Martin and Thomas, 1981; Seipp et al., 2010; Piraino et al., 2011; Mayorova and Kosevich, 2013). By contrast, the hydrozoan *Podocoryne carnea* shows a tyrosinated tubulin-LIR nerve net that forms in aboral-oral direction, as well as RFa-LIR sensory cells in the mid-body region of the early larva and in the posterior body region of older larvae (Gröger and Schmid, 2001).

Cubozoan planula larvae of *Tripedalia cystophora* show a very simple organization and appear to lack neurons. However, single ocelli are found in the posterior half of the larval body, which seem to operate as independent sensory-motor units (Nordström et al., 2003). This body plan is different to other cnidarian clades, which suggests a secondary simplification in Cubozoa.

In the basally branching Anthozoa, planulae have neurons and a nerve net that are distributed over the whole body axis. In addition, these are associated with an apical sensory organ at the aboral pole (Chia and Koss, 1979). In *Nematostella vectensis*, the apical organ consists of apical tuft cells, gland cells, and sensory cells (Zang and Nakanishi, 2020). It is sometimes considered homologous to the apical organ of marine bilaterians (Sinigaglia et al., 2015). In *Acropora millepora* and *Nematostella vectensis* planulae, a higher concentration of FMRFa-LIR neurons in the aboral half is present (Hayward et al., 2001; Nakanishi et al., 2012). This suggests that an aborally concentrated nervous system, including sensory cells with an increasing degree of aboral concentration, was present in the last common ancestor of Anthozoa, Hydrozoa, and Scyphozoa. The apical organ also seems to be a conserved structure of cnidarian planulae, since it is present in Anthozoa, the sister group to all other cnidarians (Medusozoa), as well as in the Scyphozoa (Nakanishi et al., 2008).

In the metamorphosing larva of *Cassiopea xamachana,* the FMRFa-LIR aboral nervous system degenerates at settlement. During this degeneration, supposedly apoptotic fragments from the FMRFa-LIR nervous system are found in the endoderm. After this, no ectodermal FMRFa-LIR elements can be observed until the orally concentrated nervous system of *C. xamachana* polyps develops. The first signs of the oral nervous system appear in the tentacles, where FMRFa-LIR components in the endoderm of the tentacle anlagen are present for a short period. Notably, these are replaced by ectodermal tentacle neurites shortly thereafter. Our findings point toward a loss of the planular nervous system and the *de novo* formation of the polyp nervous system. This is in accordance with the differentiation of the oral-aboral axis from larva to polyp (Sukhoputova et al., 2019). In the scyphozoan planula of *Aurelia aurita,* the aborally concentrated nervous system is also lost, most likely by apoptosis (Yuan et al., 2008), and an orally concentrated FMRFa-LIR nervous system forms *de novo* (Mayorova et al., 2012). Similar to our results, FMRFa-LIR apoptotic fragments are taken up by the endoderm during the loss of the planular nervous system (Nakanishi et al., 2008). Similar processes are present in the hydroids *Hydractinia echinata* and *Clava multicornis*, where GLWamide- and RFamide-positive cells undergo programmed cell death during metamorphosis, including the posterior neurons, while the concentrated oral nervous system of the polyp forms *de novo* (Seipp et al., 2010; Pennati et al., 2013). In planulae of the anthozoan *Nematostella vectensis,* the RPamide-LIR aboral nerve net degenerates by apoptosis and RPamide-LIR sensory cells first appear in the developing tentacles of the polyp (Zang and Nakanishi, 2020). This supports an ancestral changing of the oral-aboral polarity of the nervous system from larva to polyp.

The FMRFa-LIR and tyr. tubulin-LIR nervous system of the polyp of *Cassiopea xamachana* consists of two parts: (1) neurites lining muscle elements all over the body and (2) a loose concentration of circular neurites around the oral disc (Khabibulina and Starunov, 2020). The oral nerve ring is thought to be the integration center, while the other neurites, which innervate the muscular elements, are comparable to the motor nerve net described for scyphomedusae (Satterlie and Eichinger, 2014; Zimmerman et al., 2019; Khabibulina and Starunov, 2020). The scyphozoan *Aurelia aurita* shows a similar loose concentration of circumhypostomal neurites (Chia et al., 1984).

Compared to Scyphozoa, other cnidarian clades have more densely packed neurites, which form a single continuous nerve ring (Khabibulina and Starunov, 2020). In hydrozoan species (e.g., *Hydra*), circumhypostomal nerve rings are common (Koizumi, 2007). In *Hydra vulgaris*, two alpha-tubulin immunoreactive nerve rings are present: a proximal one with clusters of neural cell bodies (ganglion cells, sensory cells) beneath the tentacle bases, and a distal nerve ring that is more loosely organized. The latter is located halfway up the hypostome and is connected to three types of sensory cells within the hypostome ectoderm (Hufnagel and Kass-Simon, 2016). In the cubopolyp of *Tripedalia cystophora,* an ectodermal and an endodermal pair of nerve rings are present between the tentacles and the oral cone (Chapman, 1978). Also for the anthozoan *Nematostella vectensis* a circumhypostomal nerve ring has been reported (Marlow et al., 2009). Thus, an orally concentrated nerve net is most likely part of the ground pattern of cnidarians. In contrast to other cnidarian polyps (Martin, 2000; Marlow et al., 2009; Mayorova et al., 2012; Pennati et al., 2013; Hufnagel and Kass-Simon, 2016), anti-FMRFamide and anti-tyrosinated tubulin antibodies only stain neural processes but not neural somata in *C. xamachana* polyps.

In the *Cassiopea xamachana* planula, serotonin-LIR neurons appear to be absent. The first serotonin-LIR was detected in hypostomal cells of the tentacle stage. In *C. xamachana* polyps, the endodermal cells of the tentacles show serotonin-LIR components within their somata, but no serotonin-LIR processes could be detected. Thus, their cell type and putative function remain obscure. The scyphozoan *Aurelia aurita* and some hydrozoan species (*Phialidium gregarium*, *Eudendrium racemosum*, *Gonothyraea loveni*) show serotonin-LIR cells in the aboral (anterior) planula ectoderm prior to metamorphosis (McCauley, 1997; Zega et al., 2007; Mayorova et al., 2014). In *Aurelia aurita,* the aboral serotonin-LIR cells (supposedly colocalized with the FMRFamide-LIR apical organ cells) and the serotonin-LIR lateral ectodermal cells in the anterior half may constitute gland cells, epitheliomuscular cells, or lateral neurons (Mayorova et al., 2014). In the anthozoan *Aiptasia pallida*, serotonin-LIR neurites are present in the tentacles (Westfall et al., 2000).

### Evolutionary considerations

Our results support a ground pattern of cnidarian planulae with a neural plexus containing ectodermal sensory cells and mostly longitudinally oriented neural fibers. The aboral concentration of this planula nervous system seems to have increased after the anthozoan-medusozoan split. With respect to Hydrozoa, the aboral concentration further increased in Scyphozoa, with sensory cells situated only in the aboral half in semaeostome and rhizostome scyphozoan planulae. However, comparative data for the third scyphozoan clade, Coronatae, are still lacking. In *Cassiopea xamachana*, the nervous system undergoes considerable reorganisation during the planula-to-polyp transition. The larval nervous system degenerates entirely and the orally concentrated nervous system of the polyp forms *de novo*. This condition has also been described for Anthozoa as well as the medusozoans Hydrozoa, Scyphozoa, and Cubozoa. It was thus most likely present in the last common ancestor of Cnidaria. Furthermore, the polyp nervous system shows an oral concentration, which is also considered a conserved feature of Cnidaria. Concerning the scyphopolyp bodyplan, our results support a loose, ring-like concentration of the nerve net around the oral disc. This feature is less distinct than in other cnidarian clades, such as Anthozoa, Hydrozoa, and Cubozoa, which exhibit a condensed ring nerve. Accordingly, this lesser concentrated nerve net found in rhizostome and semaeostome scyphozoans most likely constitutes a secondary simplification in Scyphozoa.

Notably, our comparisons of *C. xamachana* sexual and asexual life cycle stages reveal striking similarities. We therefore conclude that the neuromyogenic programs of the planula larva were largely coopted into planuloid bud development. The only exception to this is that planuloids receive a headstart on septal muscle and neurite formation, because they partially inherit these structures from the budding (mother) polyp. Serotonin-LIR cells, which are absent from the budding zone of the *C. xamachana* polyp, appear at much earlier stages in planuloids than in planulae, suggesting that pre-specified progentiors might have been inherited instead.

## Data availability statement

The original contributions presented in the study are included in the article/supplementary material, further inquiries can be directed to the corresponding author.

## Ethics statement

Ethical approval was not required for the study involving animals in accordance with the local legislation and institutional requirements because no restrictions on the animals used here apply.

## Author contributions

KA: Formal analysis, Investigation, Methodology, Visualization, Writing – original draft. EZ: Formal analysis, Investigation, Methodology, Supervision, Validation, Writing – review & editing. DA-N: Methodology, Resources, Writing – review & editing. AWe: Methodology, Resources, Writing – review & editing. AWa: Conceptualization, Funding acquisition, Formal analysis, Validation, Writing - manuscript finalization, Investigation, Project administration, Resources, Supervision, Writing – review & editing.
